# Impact of Select Geometric and Operational Parameters on Hydrodynamics in Dissolution Apparatus 2 (Paddle Apparatus): A Design of Experiments Analysis Based on Computational Fluid Dynamics Simulations

**DOI:** 10.1007/s11095-022-03272-4

**Published:** 2022-05-16

**Authors:** Satish Perivilli, Steven Walfish, Erika Stippler, Mark R. Liddell

**Affiliations:** 1 US Pharmacopeial Convention, 12601 Twinbrook Parkway, Rockville, Maryland 20852-1790 USA; 2 European Directorate for the Quality of Medicines and Healthcare, 7 Allee Kastner, 67000 Strasbourg, France

**Keywords:** design of experiments, dissolution apparatus 2, hydrodynamics

## Abstract

**Purpose:**

A Design of Experiments (DOE) analysis driven by Computational Fluid Dynamics (CFD) simulations was used to evaluate individual and two-factor interaction effects of varying select geometric and operational parameters on the hydrodynamics in dissolution apparatus 2 (paddle apparatus).

**Methods:**

Simulations were run with meshing controls and solution strategies retained from a mesh-independent validated baseline model. Distance between vessel and impeller bottom surfaces, impeller offset, vessel radius and impeller rotation speed were considered as input parameters. The velocity magnitudes at four locations near the vessel bottom surface were considered as output parameters. Response surfaces and Pareto charts were generated to understand individual and two-factor interaction effects of input parameters on the output parameters.

**Results:**

Impeller offset has a dominating influence of a linear and quadratic nature on the output parameters and affects overall hydrodynamics. Changes to other input parameters have limited influence on velocity magnitudes at locations closest to the vessel axis and on overall hydrodynamics. However, these parameters have important influences of varying degrees on velocity magnitudes at locations away from the vessel axis.

**Conclusions:**

The hydrodynamics in Apparatus 2 is influenced differently by different parameters and their combinations. Impeller offset has a stronger influence when compared to parameters that do not alter apparatus symmetry.

## INTRODUCTION

Dissolution Apparatus 2, or the paddle apparatus (referred to as App 2 from here on), is a dissolution apparatus described in USP General Chapter < 711 > *Dissolution* (1) that is widely used in testing of the dissolution behavior of a multitude of drug products. The apparatus consists of a hemispherical-bottomed cylindrical vessel that contains the dissolution medium, and an impeller (a connected paddle-shaft combination) that rotates within the vessel at a specified speed. Typical dissolution testers (apparatus assemblies) contain 6–8 vessels with corresponding impellers. In each vessel, a dosage form is placed ideally at the bottom center directly under the impeller. Variability in dissolution results could arise from differences in hydrodynamic conditions that the dosage forms are subjected to. Assuming same position of the dosage form in each vessel, the different hydrodynamic conditions could indicate differences in either geometric or operational setup within the assembly. Thus, understanding the hydrodynamics in App 2 operating under different conditions is critical and consequently, has been a subject of sustained research over the past few decades.

In one of the earliest investigations of App 2 hydrodynamics, Bocanegra *et al.* (2) measured fluid velocity components, in specific regions of interest, at two different paddle^1^ rotation speeds using an Experimental Fluid Dynamics (EFD) method. More recent works have employed both EFD methods and CFD simulations to understand the hydrodynamics in App 2 (3–8). While it is necessary to validate CFD results with EFD data, CFD simulations provide holistic information on hydrodynamics within the computational domain and also provide a relatively easy platform for parametric studies. Literature describing baseline CFD models is readily available (7,8). These CFD models were also extended, by the respective groups, to understand the effects of individual operational (i.e., impeller rotation speed (9,10)) and geometric parameter (i.e., impeller location (11)) variations on App 2 hydrodynamics.

While there is considerable interest in understanding the changes in App 2 hydrodynamics with varying parameters, there are no studies known that investigated the influence of combinational parametric changes. This paper presents the results from a CFD simulation driven DOE analysis over various vessel-impeller combinations. As the focus was on understanding the changes in hydrodynamics that result from design and/or operational changes to the apparatus, the presence of a dosage form was not considered in the simulations. Thus, analysis of potential effects of various hydrodynamic characteristics on the dissolution behavior of a dosage form is outside the scope of this paper.

Tools available within the ANSYS® WorkBench™ (ANSYS 16.2) platform were used for carrying out the simulations required for this study.

## CFD AND DOE METHODS

### Design Space for the DOE

For the purposes of this study, the distance between vessel and impeller bottom surfaces (d), the impeller offset, i.e. centering (pdl_off), the vessel radius (R) and impeller rotation speed (Ω) were considered input parameters. The values considered for these were defined based on (1) and are presented in Table I. ANSYS® DesignXplorer™ was used to generate the design table, presented in Table II (only input parameters shown). A standard face-centered Central Composite Design (CCD) was used to design this table.

**Table I:** Low, Center and High Values of Input Parameters

Parameter No	Parameter Description	Units	Low	Center	High
P1	Distance between vessel and impeller bottom surfaces, d	mm	23	25	27
P2	Impeller offset, pdl_off	mm	0	1	2
P3	Vessel radius, R	mm	49	51	53
P4	Impeller rotational speed, Ω^a^	rpm	-52	-50	-48

^a^ The negative rotational speeds indicate rotation in clockwise direction per ANSYS® FLUENT^TM^ specifications. This direction was retained to match with results shown in (7). In actuality, Ω = -52 rpm represents the impeller rotating at a greater speed than, for example, Ω = -48 rpm

**Table II:** Simulation Design Table

Run No	P1 (d, mm)	P2 (pdl_off, mm)	P3 (R, mm)	P4 ( Ω, rpm)
1	25	1	51	-50
2	23	1	51	-50
3	27	1	51	-50
4	25	0	51	-50
5	25	2	51	-50
6	25	1	49	-50
7	25	1	53	-50
8	25	1	51	-52
9	25	1	51	-48
10	23	0	49	-52
11	27	0	49	-52
12	23	2	49	-52
13	27	2	49	-52
14	23	0	53	-52
15	27	0	53	-52
16	23	2	53	-52
17	27	2	53	-52
18	23	0	49	-48
19	27	0	49	-48
20	23	2	49	-48
21	27	2	49	-48
22	23	0	53	-48
23	27	0	53	-48
24	23	2	53	-48
25	27	2	53	-48

**Fig. 1:**
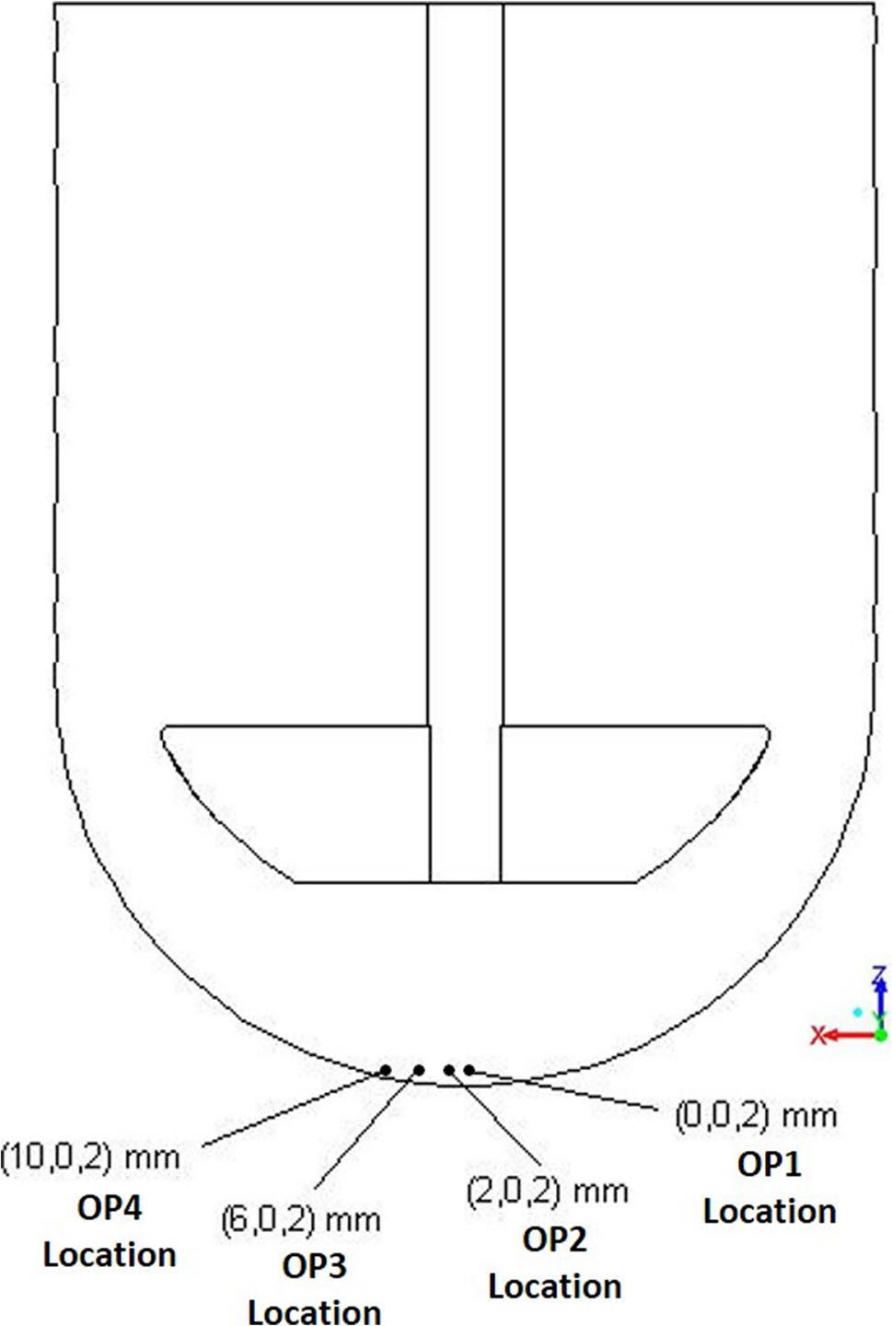
Location of points related to the output parameters; position coordinates (x,y,z) shown.

The influence of the input parameter values was investigated by examining the changes to four output parameters (OP1 through OP4) defined to be the iteration averaged vertex averages of velocity magnitudes at specific locations. The averaging methods are described later in the Solution sub-section under Development of CFD Model(s) section. These locations were chosen to be near the bottom of the vessel as this region is considered to be of interest in App2, since this is the region where a solid dosage form is typically located. The four selected locations are shown in Fig. 1.It is important to note that, in this paper, the positions of the four locations are always referenced to the fixed vessel axis irrespective of any changes to geometry considered within the DOE cases.

### Development of CFD Model(s)

In preparation of the DOE analysis, it was first essential to establish a validated (mesh-independent) baseline solution^2^ for App 2 hydrodynamics to finalize the choices of meshing controls and solution strategies. These were retained across all simulations for the DOE runs. The development of the baseline solution, with its implications on the DOE runs, is described in the following text. As the focus of the current study was to describe the flow of liquid medium contained in the vessel, the geometry of the model(s) was restricted to the liquid domain only. The liquid was considered to be water at 37°C.

#### Geometry

The geometric and operational parameters employed for the baseline case are presented in Table III. In general, all geometric dimensions were within specifications defined in (1). However, values for some of the parameters such as the vessel diameter, the impeller rotational speed and the liquid volume in vessel were chosen from (7) specifically to validate the CFD model with Laser Doppler Velocimetry (LDV) data presented therein. For the purposes of this study, the radius of the hemispherical bottom part of the vessel was considered to be equal to the radius of the cylindrical part. Also, as a modeling simplification, the blend between the paddle and shaft was not considered to avoid complexities with mesh generation. It is assumed that this slight geometrical difference would not impact overall hydrodynamics significantly.

**Table III:** Geometrical and Operational Parameters Used for the Baseline Model

Parameter	Value	Reference
Paddle height, h_p_	19.0 mm	(1)
Paddle “bounding edge” radius	41.5 mm	(1)
Paddle “bounding edge” center location from paddle bottom	35.8 mm	(1)
Paddle thickness, t_p_	4.0 mm	(1)
Paddle upper diameter, Φ_p,u_	75.0 mm	(1)
Paddle lower diameter, Φ_p,l_	41.981 mm^b^	-
“Bounding edge” blend radius at paddle corners	1.2 mm	(1)
Distance between impeller and vessel bottom surfaces, d	25.0 mm	(1)
Shaft diameter, Φ_sh_	9.5 mm	(1)
Impeller offset, pdl_off	0 mm	(1)
Liquid volume, V	900 mL	(7)
Vessel diameter, Φ_v_	100.16 mm	(7)
Impeller rotational speed, Ω	50 rpm	(7)

^b^ measured from geometry of model and checked to be within specifications of (1)

**Fig. 2:**
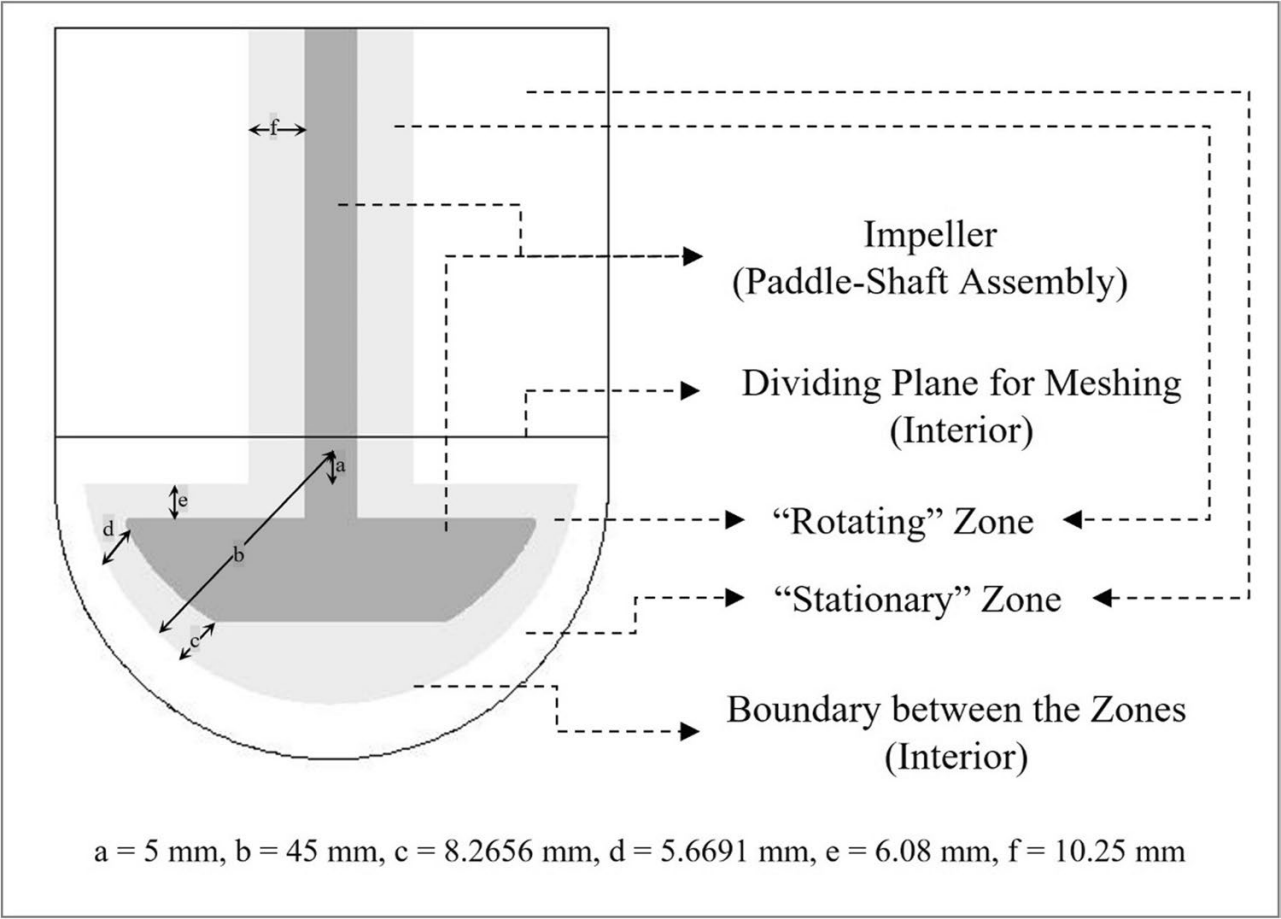
Geometry used for baseline simulation.

The computational domain height (liquid level), required to complete the geometry, was determined based on liquid displacement calculations that account for the submerged impeller in the liquid. A three-dimensional geometry that represents App 2 was built using ANSYS® DesignModeler™. Figure 2 shows a depiction of the geometry of the computational domain used. A moving reference frame methodology was used to limit computational expenses; the use of a moving reference frame enables steady-state solution (in a moving frame) of an unsteady-state problem (in a stationary frame) (13). For this reason, the geometry was divided into two zones to be modeled as rotating and stationary zones, using an “interior” boundary between the zones. The hemispherical region of the interior boundary was designed with its center located 5 mm above the top surface of the baseline geometry’s hemispherical part of the vessel (parameter a) and having a radius of 45 mm (parameter b in Fig. 2). The 45 mm was chosen to ensure the reference frame boundary is close to being parallel to the curvature of the vessel hemispherical bottom for the baseline model (when viewed from the viewpoint shown in Fig. 2). For the DOE cases, the location of this origin was adjusted based only on changes to impeller offset and/or distance between paddle and vessel bottom surfaces (but not with changes to vessel radius) to maintain the relative position of the reference frame to the impeller. In other words, the rotating zone was designed to adaptively change with geometric changes arising from changes to input parameters such that its relative position to the rotating impeller was maintained for all geometries. This also ensured that the rotating zone remained a body of revolution centered around the impeller axis. In all cases, the axis of rotation for the rotating zone coincided with that of the impeller.

#### Meshing

For meshing convenience, the rotating and stationary zones were further divided into upper and lower regions using a dividing “interior” plane as shown in Fig. 2. Sweep methods that create hex/wedge elements were used in the upper regions. Using hex/wedge elements reduces the overall element count and thereby, leads to reduced simulation time. The upper region was designed to encompass 90% of the cylinder height as measured from the top surface. The remaining part of the domain was meshed automatically. The 90% length was chosen to retain the proportion of the sweep meshed region even with changes to the moving reference frame location for the different DOE cases.

**Fig. 3:**
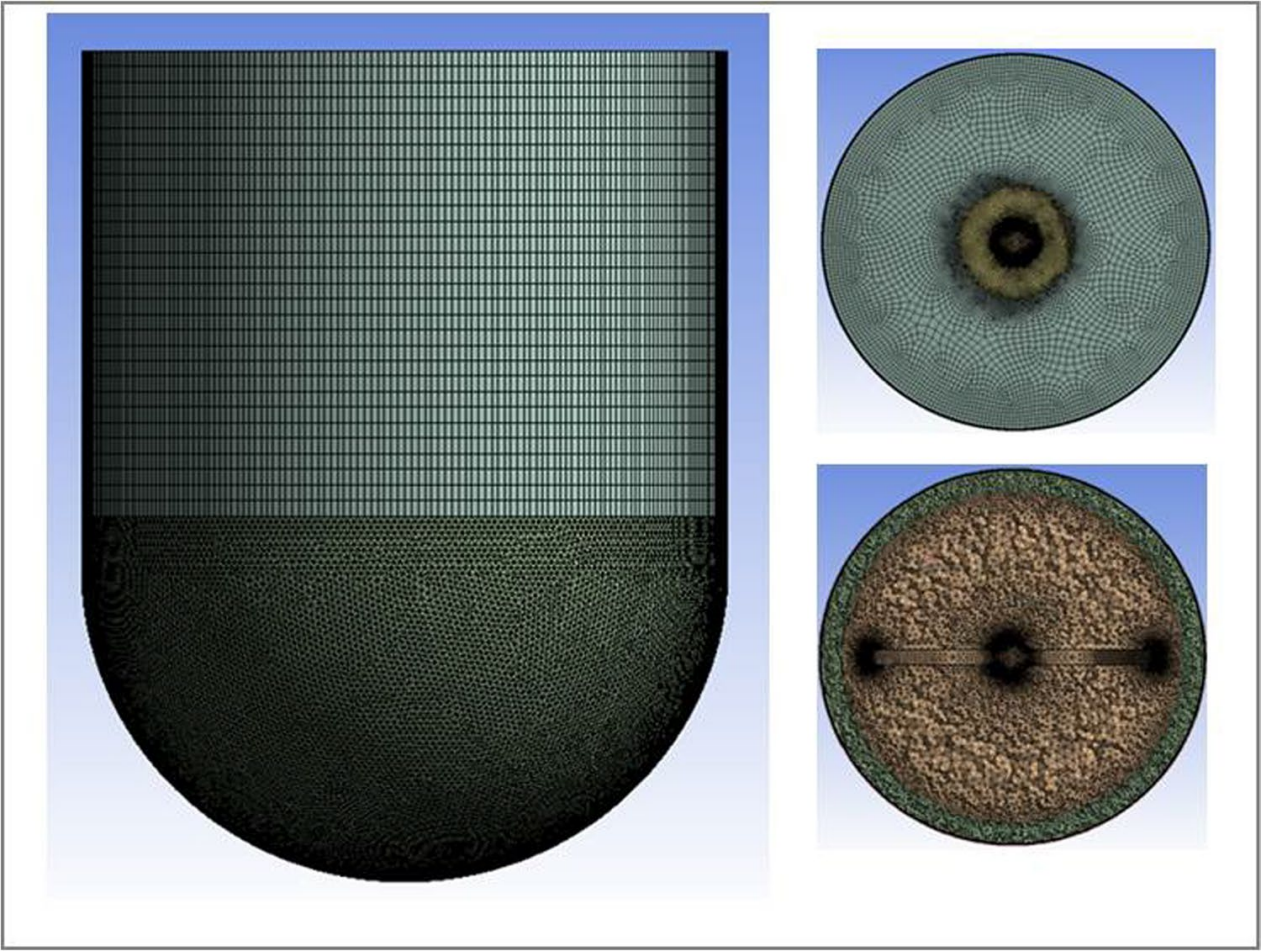
Mesh from the (mesh-independent) baseline solution.

For the baseline case, the computational mesh was generated with advanced size functions on curvature globally. Additionally, inflation controls were applied along the impeller and vessel walls for local refinement. Results from five different meshes, with progressively increasing number of elements, were evaluated for mesh independence. Figure 3 shows the mesh, which yielded the mesh-independent solution, for the baseline case along with two cross sections to show inflation along the vessel and impeller walls. Meshes for DOE cases, with varying geometries, were generated using the meshing controls from the baseline mesh-independent solution. For DOE cases that shared geometry, the first generated meshes were re-used for consistency.

#### Solution

A three-dimensional double precision pressure-based solver with relative velocity formulation was used for all simulations reported here. For all cases presented here, the region depicted as “rotating” zone in Fig. 2 was defined with a frame motion condition of rotation and the speed was adjusted as needed. For the baseline case, the rotation speed was specified to be -50 rpm to be consistent with (7). In all cases, the rotation-axis origin was specified to lie on the impeller axis (with the geometry already modified with re-positioning of the rotating zone, as required) and the axis of rotation was defined to be the vector from the rotation-axis origin in the requisite direction. The impeller walls were specified as boundaries with zero wall motion relative to the moving reference frame with the rotation origin. The region outside the rotating zone was defined as being stationary at initialization while the vessel wall was defined to be stationary as a boundary condition. The top surfaces were defined with symmetry boundary conditions.

For all cases, steady-state solutions were obtained using the SST $\upkappa -\upomega$ model with the “Production Limiter” option enabled for the viscous model. Coupled scheme was used for the pressure–velocity coupling while the least squares cell based scheme was used for gradient calculations. The equation for pressure was discretized using PRESTO! while equations for momentum, $\mathrm{\kappa \,\small and \,} \normalsize \upomega$ were all discretized using second order upwind schemes. For solution, pseudo transient calculations were enabled with the automatic time step method and a time scale factor of 2. Solutions were initialized with hybrid initialization option and subsequently a better initial solution was provided by employing the Full Multigrid (FMG) initialization (13).

For each simulation, residual and surface monitors were used to monitor convergence. As the focus of the proposed DOE study was on evaluating the changes to previously defined output parameters, these were also included in the surface monitors for assessment. In situations where the output parameter surface monitors converged to a value (as identified by the difference in maxima and minima, over the last quarter of iterations, being less than or equal to 0.0005 m/s), the average over the last one-tenth iterations was used for the value of the corresponding velocity magnitude. In other situations, where the monitors converged with cyclic/wavy variation (i.e., when the difference was greater than 0.0005 m/s), the velocity magnitudes, at such locations, were determined to be the average value of the last full cycle.^3^ Certain DOE cases were run for more iterations than others to ensure that the surface monitors converged to either one of the two states described. The averaged output parameter values were used in subsequent statistical analysis. However, it should be noted that all contour and vector plots presented in the following sections are from the final iterations irrespective of the convergence behavior.

### Statistical Analysis

Upon completion of all DOE runs, a statistical analysis was performed using Minitab® 18 to investigate individual and two-factor interaction effects of input parameters on the output parameters. Analysis of Variance (ANOVA) tables and models were generated for each output parameter to determine if the different factors were statistically significant. Any effect whose p-value was more than 0.10 was removed during model fitting. A p-value of less than 0.05 was considered to be statistically significant. Pareto charts that showed the absolute values of the standardized effects from largest to smallest were also generated.

## RESULTS

**Fig. 4:**
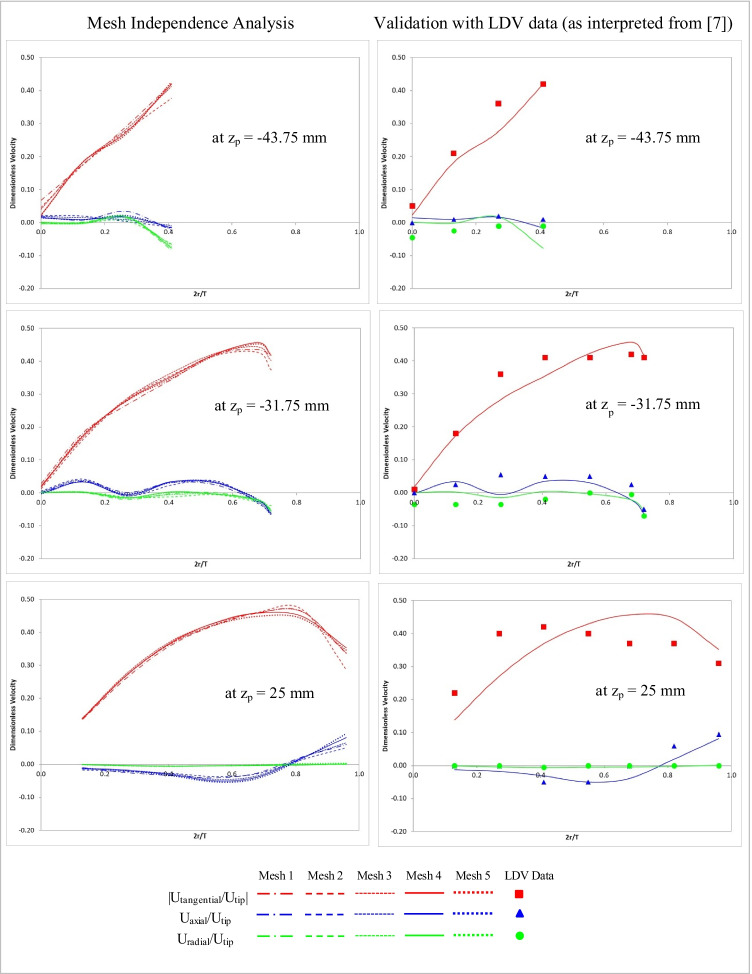
Mesh independence analysis and validation of baseline results.

Baseline results from the five different meshes were compared for mesh-independence and validation against LDV data as interpreted from literature (7) (minor differences in geometries and comparison locations between (7) and the baseline case presented here notwithstanding). For these purposes, definitions of U_tip_ and z_p_ (in that z_p_ = 0 represented the intersection of the hemispherical and cylindrical parts of the vessel) were retained from (7) despite small differences in vessel radius, R between the two studies. Additionally, as was done in (7), component values, corresponding to eight different azimuthal locations were averaged to yield a single value for a radial and axial coordinate combination. Comparisons at selected locations are shown in Fig. 4. The mesh independence analysis and validation are presented separately and respectively as images to the left and right at any given location. In general, results from Mesh 4 and Mesh 5 were found to be closely following each other with Mesh 4 results also comparing favorably with LDV data (7). Thus Mesh 4 was regarded as providing a mesh-independent and validated solution for the baseline case. Mesh 4 consisted of about 5.5 million elements with a maximum skewness value of 0.86. Moving forward, meshing controls from Mesh 4 were used to generate results for all DOE cases. Consistent with observations of (7), for App 2, the tangential component of velocity was found to be the dominating component of velocity.

**Fig. 5:**
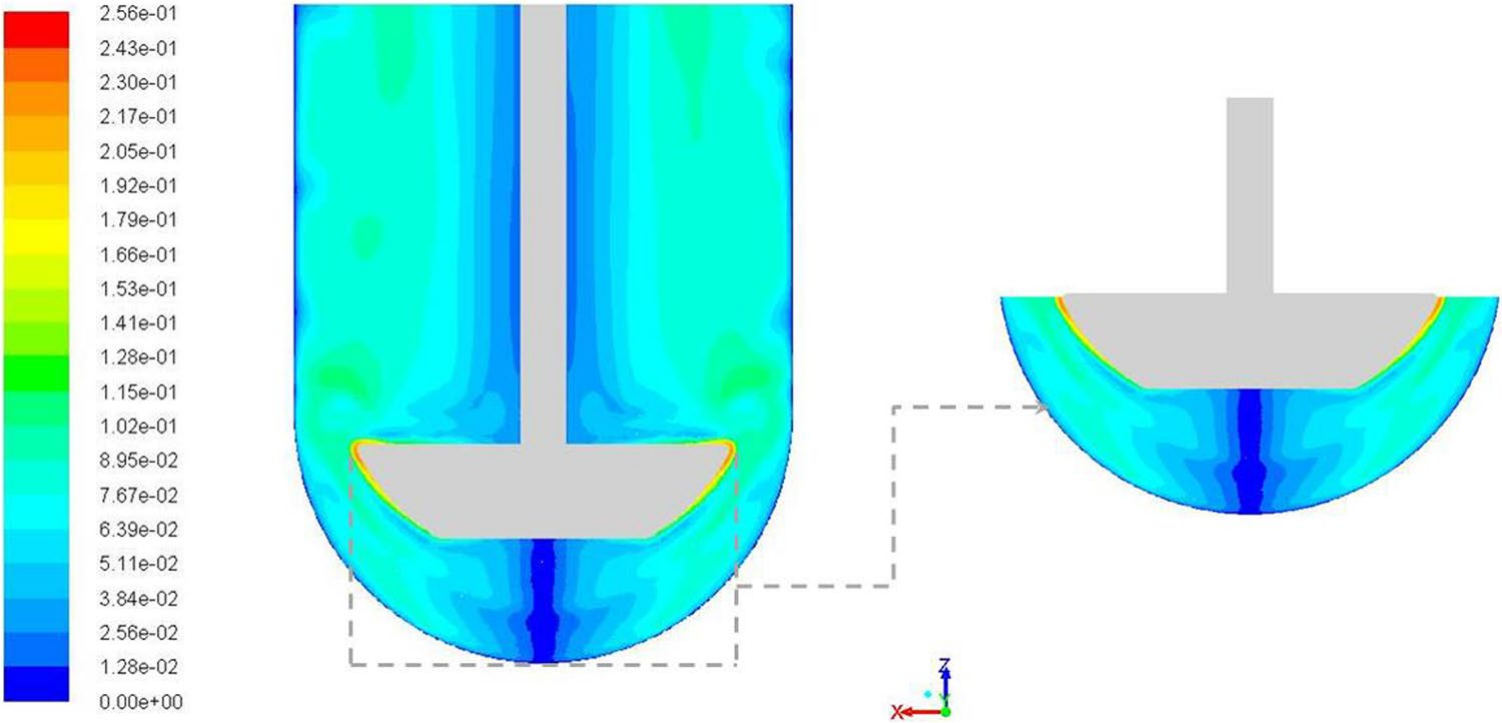
Velocity magnitude (m/s) contours along a vertical central plane with emphasis on the region below the impeller.

Figure 5 shows the velocity magnitude contours along a central vertical plane for the baseline case. While the velocities were high near the paddle tip, there is a decrease in velocity magnitudes with increasing distance in the radial direction towards the vessel wall. The no-slip condition dictates that the velocity along the vessel wall is zero. In the region above the paddle, there are low velocity regions around the shaft and the vessel wall surfaces. In between these two low velocity regions, comparatively higher velocity magnitudes are predicted. In the region below the paddle, there are two high velocity regions that surround a low velocity magnitude region in the central region (commonly referred to as the “dead zone”).

**Fig. 6:**
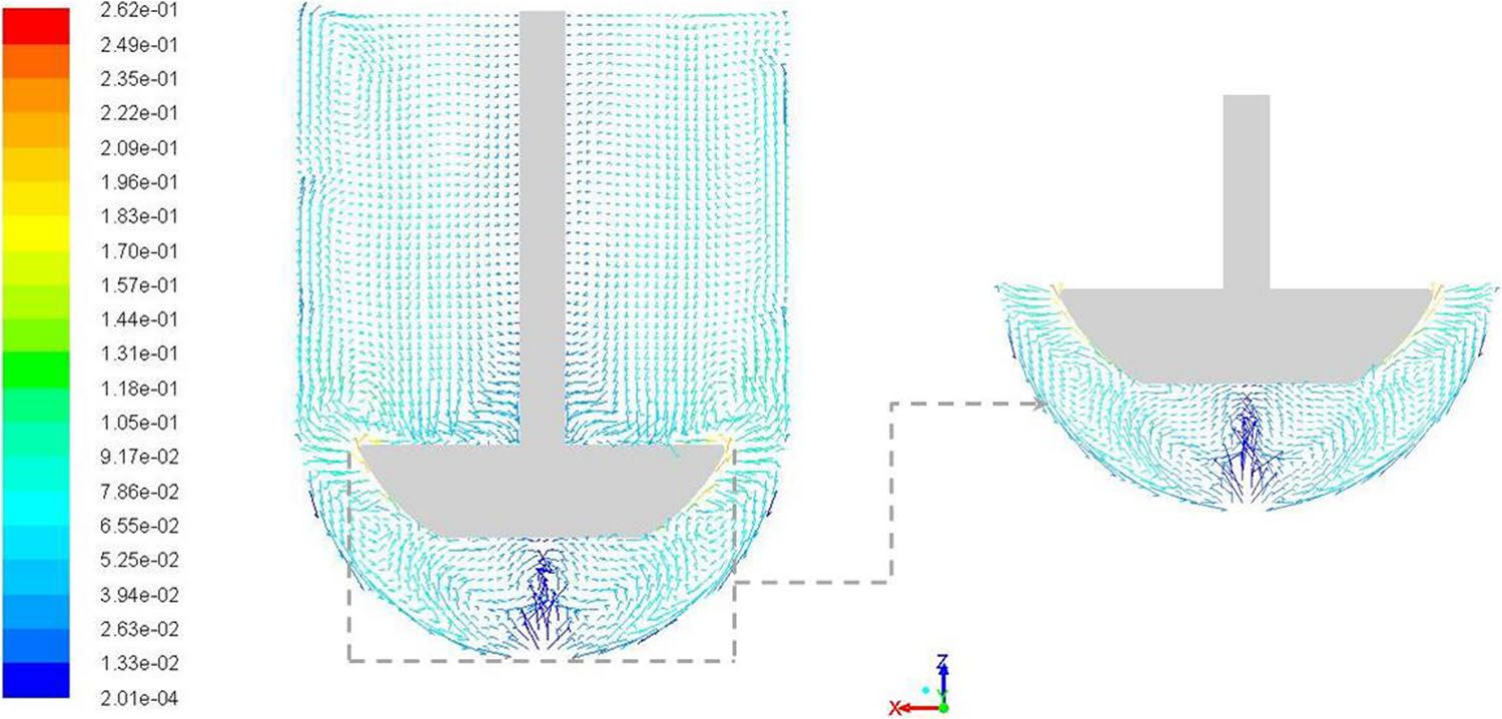
Velocity magnitude (m/s) vectors along a vertical central plane with emphasis on the region below the impeller; plane created with 100 samples in each direction; parts of picture re-used from (12) with permission from Dissolution Technologies.

Figure 6 shows in-plane vector plots along the central vertical plane for the baseline case. Flow from the paddle tip is directed in two different directions. There is one part of the flow that is directed axially upwards while the other is directed axially downwards – both along the vessel wall. The upward flow continues in that direction to the end of the fluid domain, i.e., the surface of the liquid, where it is redirected downwards towards the paddle creating a recirculation loop above the paddle. The recirculation loop is bounded by low magnitude flow near the shaft wall. The downward directed part of flow establishes a different recirculation loop below the paddle. However, a stagnant low velocity zone, i.e., dead zone, is observed directly below the paddle surface along the vessel axis as the recirculation doesn’t extend to the center. The flow phenomenon discussed here, for this central vertical plane, was observed to be symmetric about the impeller axis. The vectors are shown as fixed length vectors in the figure (with vectors that appear smaller being representative of vectors that are angled into or out of the plane) and this presentation technique is used for all vectors in this paper.

The baseline results described hitherto provide an understanding of the typical flow distribution in App 2 setup with parameters d = 25 mm, pdl_off = 0 mm, R = 50.08 mm and Ω  = -50 rpm (see Table III). The DOE runs were programmed to understand the effects of changing the input parameter values on the hydrodynamics of App 2. Even though the essential focus was on the changes in the output parameters, CFD simulation results were also analyzed to draw comparisons.

### DOE Analysis

The output parameter values were computed for each DOE case using the averaging procedure described in the preceding “Solution” sub-section. Run 10 in Table II failed to converge to a steady-state, and thus was eliminated from the DOE analysis. For each output parameter, ANOVA tables and models were generated with statistical parameters defined previously in the statistical analysis section. In the subsequent discussion, the impact of varying P1 (distance between vessel and impeller bottom surfaces, d), P2 (impeller offset, pdl_off), P3 (vessel radius, R) and P4 (impeller rotation speed, Ω) on the output parameters is presented.

#### Influence of Input Parameters on OP1

**Fig. 7:**
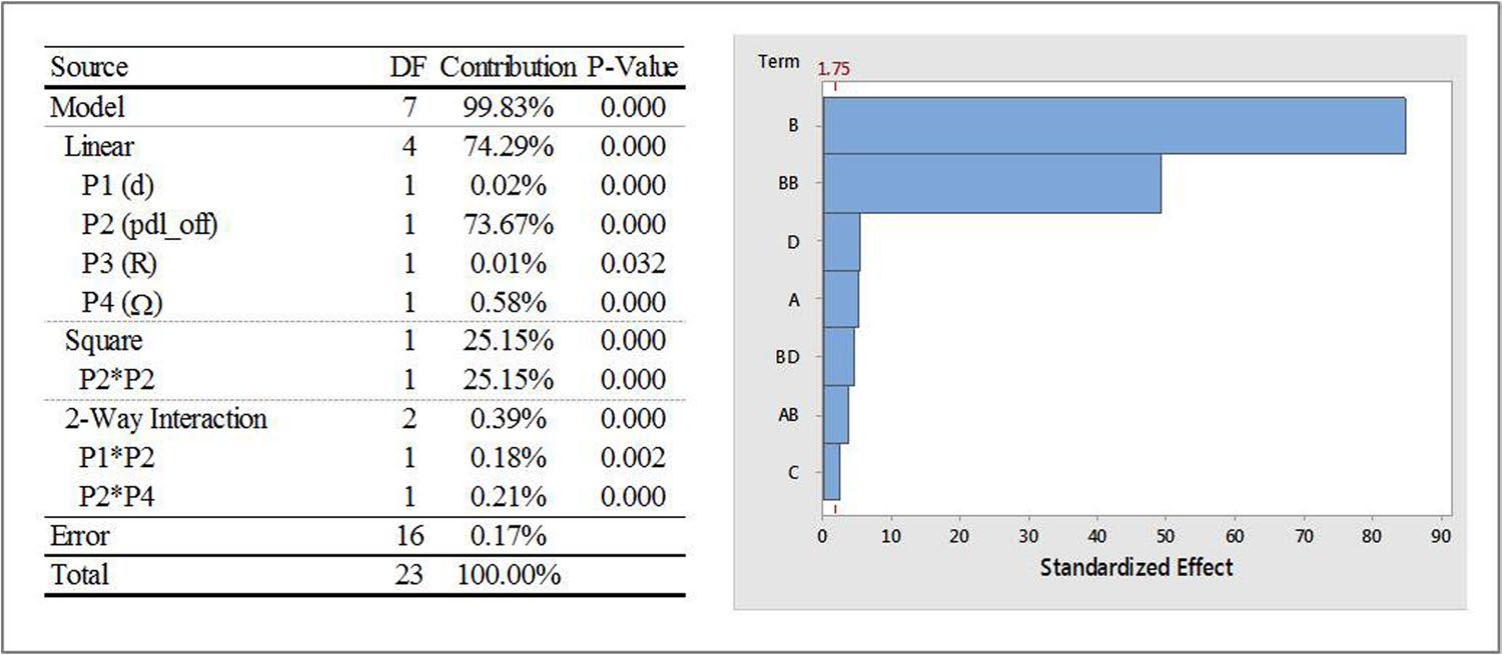
ANOVA analysis and Pareto chart of standardized effects, with α = 0.1, on OP1; Factor definitions for Pareto chart: A ↔ P1 (d), B ↔ P2 (pdl_off), C ↔ P3 (R), D ↔ P4 (Ω).

Figure 7  shows the ANOVA analysis with selected statistics, and a Pareto chart that describes the individual and two-way interaction effects of input parameters on OP1 (iteration averaged vertex average of velocity magnitude at a point location that lies on the vessel axis directly underneath the paddle). The standardized effects, shown on the x-axis of the Pareto chart, are t-statistics that test the null hypothesis that the effect is zero. The Pareto chart also shows a reference line to indicate which effects were statistically significant. It can be seen that, of the four input parameters considered, P2 (pdl_off) has the most dominating influence on OP1 and is seen to have linear, square and two-way interaction effects on OP1. In fact, the Pareto chart indicates that the two highest effects are the linear and square effects of the impeller offset. There are relatively minor, yet statistically significant linear effects of P1 (d), P3 (R) and P4 (Ω) as well as interaction effects of P2 with P1 and P4.

**Fig. 8:**
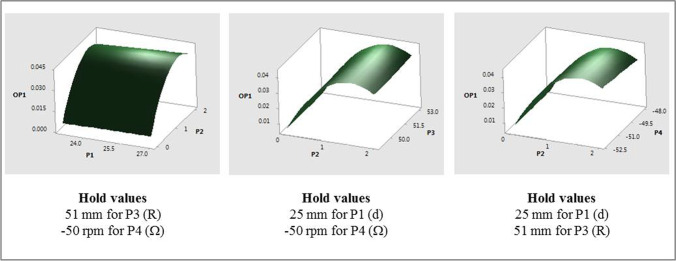
OP1 response surfaces for two-factor interactions of P2 (pdl_off).

Response surface plots that show the relationship between OP1 and various combinations of P2, with other input parameters are shown in Fig. 8. Similar response surfaces were generated for other parametric combinations but are not presented here for the sake of brevity. The individual and interaction effects of the input parameters on the fitted means of OP1 are as follows:

- The mean values of OP1 were found to change quadratically with P2 with increasing values for approximately 0 ≤ P2 ≤ 1.5 mm followed by a decrease to P2 = 2 mm. Similar trends were observed for all values of P4 studied with higher speeds resulting in higher means.
- The mean values of OP1 increased linearly (albeit slightly) with increasing values of P1, P3 and P4 (magnitude). The mean values of OP1 were comparatively lower for cases with P2 = 0 mm as compared to those with non-zero values of P2.

#### Influence of Input Parameters on OP2

**Fig. 9:**
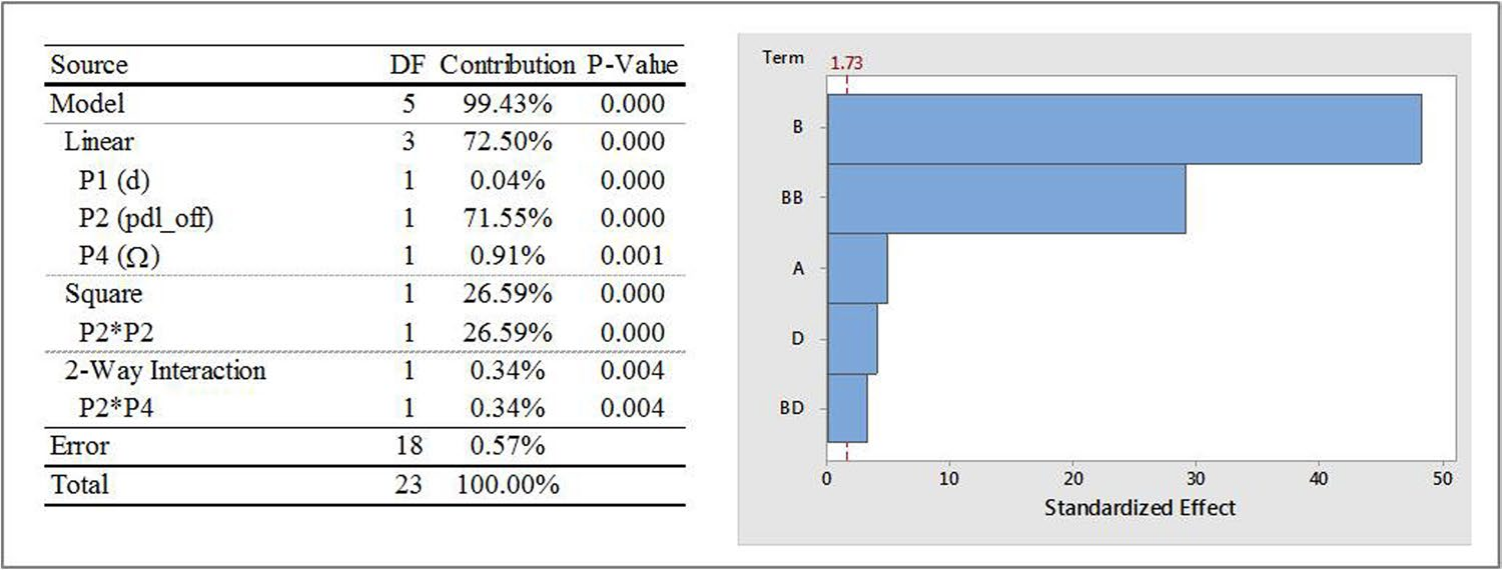
ANOVA analysis and Pareto chart of standardized effects, with α = 0.1, on OP2; Factor definitions for Pareto chart: A ↔ P1 (d), B ↔ P2 (pdl_off), C ↔ P3 (R), D ↔ P4 (Ω).

Figure 9 shows the ANOVA analysis and a Pareto chart for OP2 (iteration averaged vertex average of velocity magnitude at a point location that lies 2 mm from the vessel axis in the positive x- direction). As was the case with OP1, of the four input parameters considered, P2 (pdl_off) has the most dominating influence on OP2 and is seen to have linear, square and two-way interaction effects on OP2. The Pareto chart indicates that the two highest effects are the linear and square effects of the impeller offset. These are followed by relatively lesser, yet statistically significant, linear effects of P1 (d) and P4 (Ω) and interaction effects P2 and P4. P3 (R) was not found to have any statistically significant effects on OP2.

**Fig. 10:**
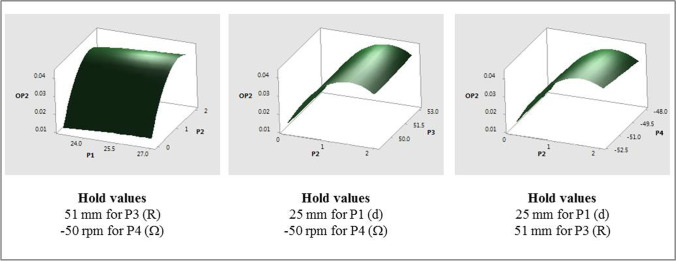
OP2 response surfaces for two-factor interactions of P2 (pdl_off).

Response surface plots that show the relationship between OP2 and various combinations of P2, with other input parameters are shown in Fig. 10. The individual and interaction effects of individual input parameters on the fitted means of OP2 are as follows:

- The mean values of OP2 were found to change quadratically with P2 with increasing values for approximately 0 ≤ P2 ≤ 1.5 mm followed by a decrease to P2 = 2 mm. The only interaction effect was between P2 and P4 and, as with OP1, similar quadratic trends, that followed the main effect of P2, were observed for all values of P4 considered.
- The mean values of OP2 increased linearly with increasing values of P1 and P4 (magnitude).

#### Influence of Input Parameters on OP3

**Fig. 11:**
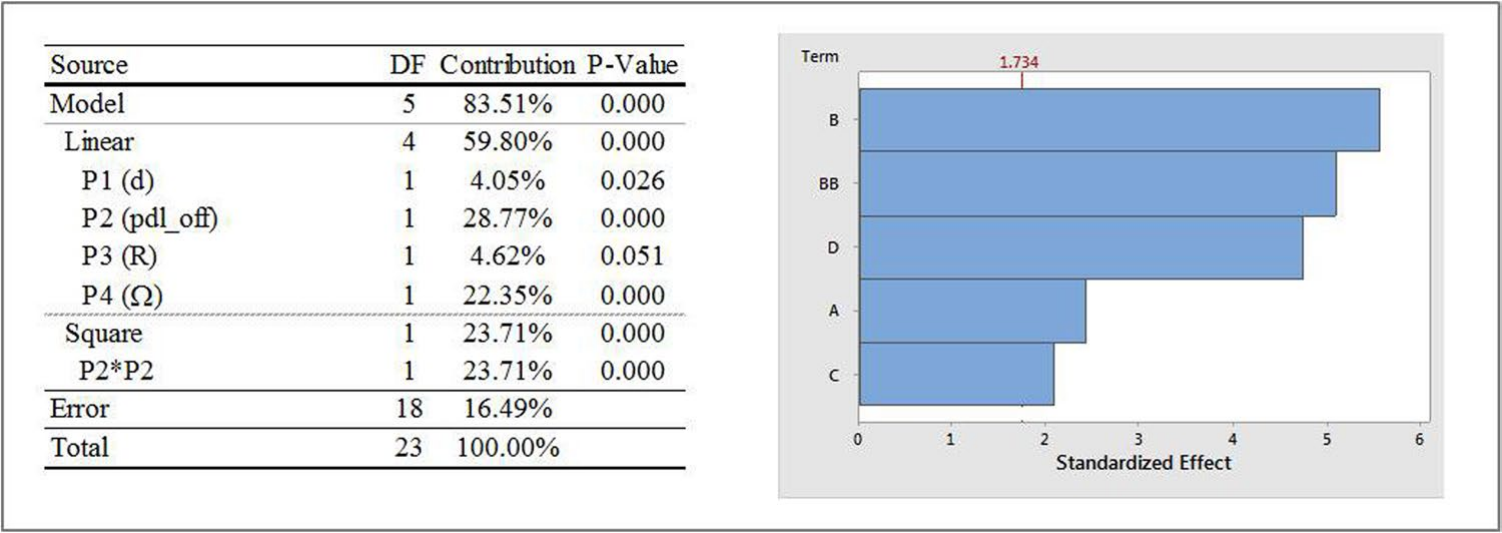
ANOVA analysis and Pareto chart of standardized effects, with α = 0.1, on OP3; Factor definitions for Pareto chart: A ↔ P1 (d), B ↔ P2 (pdl_off), C ↔ P3 (R), D ↔ P4 (Ω).

Figure 11 shows the ANOVA analysis and a Pareto chart for OP3 (iteration averaged vertex average of velocity magnitude at a point location that lies 6 mm from the vessel axis in the positive x- direction). As was the case with OP1 and OP2, of the four input parameters considered, P2 (pdl_off) has the most dominating influence on OP3. However, the effects are only linear and square in nature and even though these are the two highest effects, their combined contribution is not as high as they were with OP1 and OP2. Another noticeable difference is that the contribution of P4 (Ω) is similar in magnitude to the linear and square effects of P2 which was not the case with either OP1 or OP2. The linear effects of P3 (R) and P1 (d) are the remaining statistically significant contributors and their contributions are higher than those for OP1 and OP2. No interaction effects were seen for OP3.

**Fig. 12:**
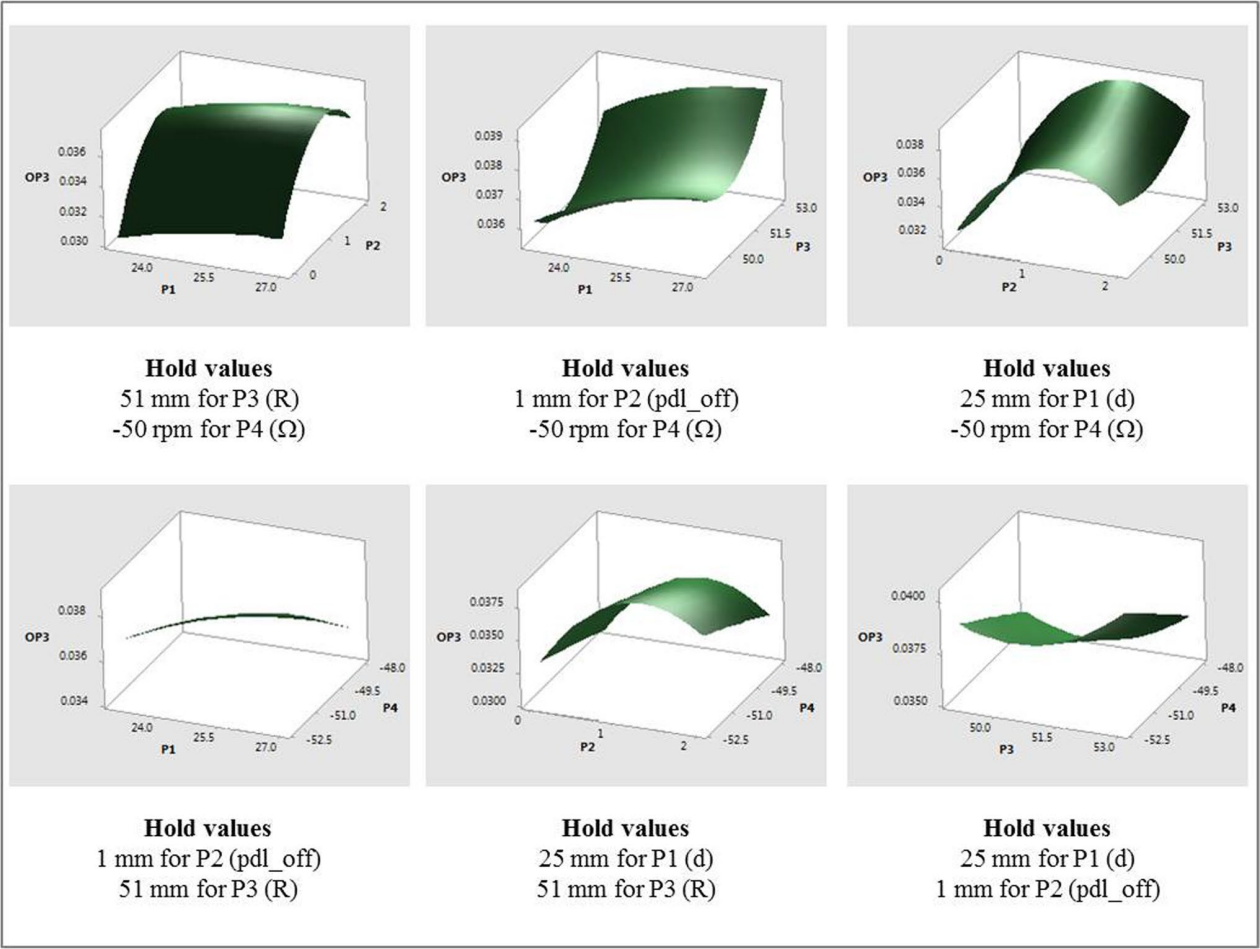
OP3 response surfaces for two-factor interactions of various input parameters.

Response surface plots that show the relationship between OP3 and various combinations of all the input parameters (due to relatively high contributions) are shown in Fig. 12. The individual effects of input parameters on the fitted means of OP3, with all other input parameters constant are as follows:

- The mean values of OP3 were found to change quadratically with P2 with increasing values for approximately 0 ≤ P2 ≤ 1.25 mm followed by a decrease to P2 = 2 mm.
- The mean values of OP3 increase with increasing values of P1, P3 and P4 (magnitude).

#### Influence of Input Parameters on OP4

**Fig. 13:**
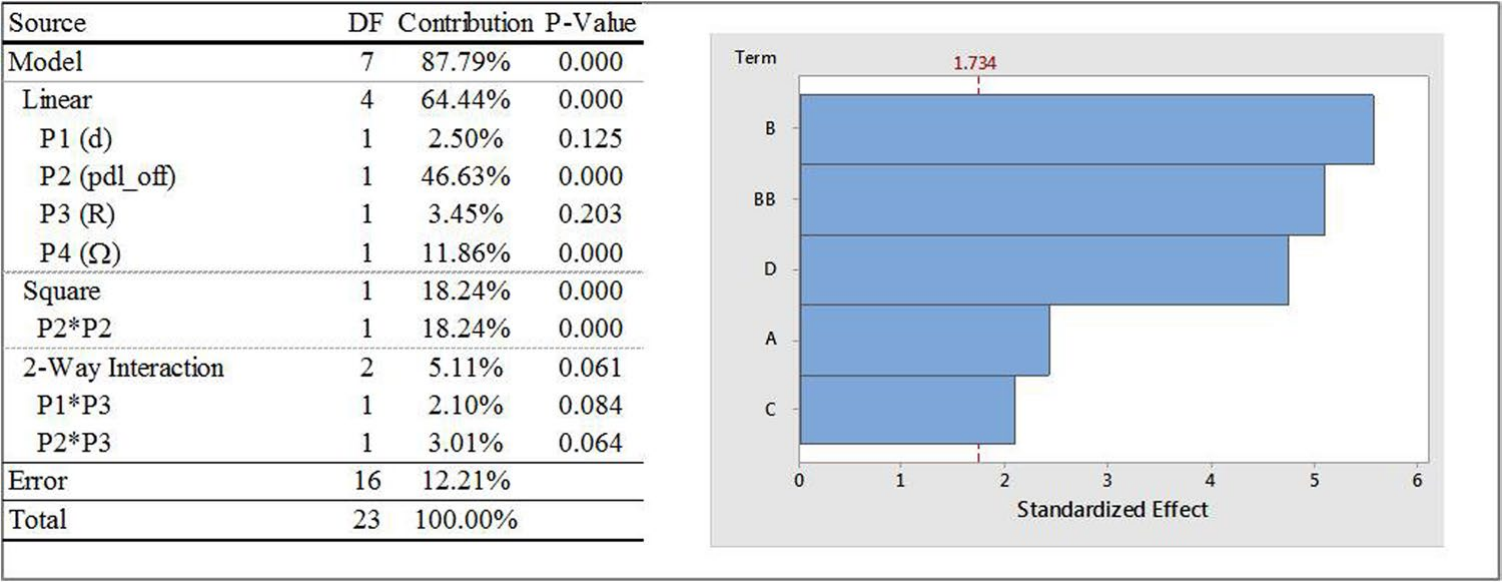
ANOVA analysis and Pareto chart of standardized effects, with α = 0.1, on OP4; Factor definitions for Pareto chart: A ↔ P1 (d), B ↔ P2 (pdl_off), C ↔ P3 (R), D ↔ P4 (Ω).

Figure 13 shows the ANOVA analysis and a Pareto chart for OP4 (iteration averaged vertex average of velocity magnitude at a point location that lies 10 mm from the vessel axis in the positive x- direction). As was the case with the other output parameters of the four input parameters considered, P2 (pdl_off) has the most dominating influence on OP4 and is seen to have linear, square and two-way interaction effects on OP4. Similar to OP3, contribution of P4 (Ω) is also high for OP4. The linear effects of P3 (R) and P1 (d) and interaction effect between P1 and P3 are the remaining statistically significant contributors.

**Fig. 14:**
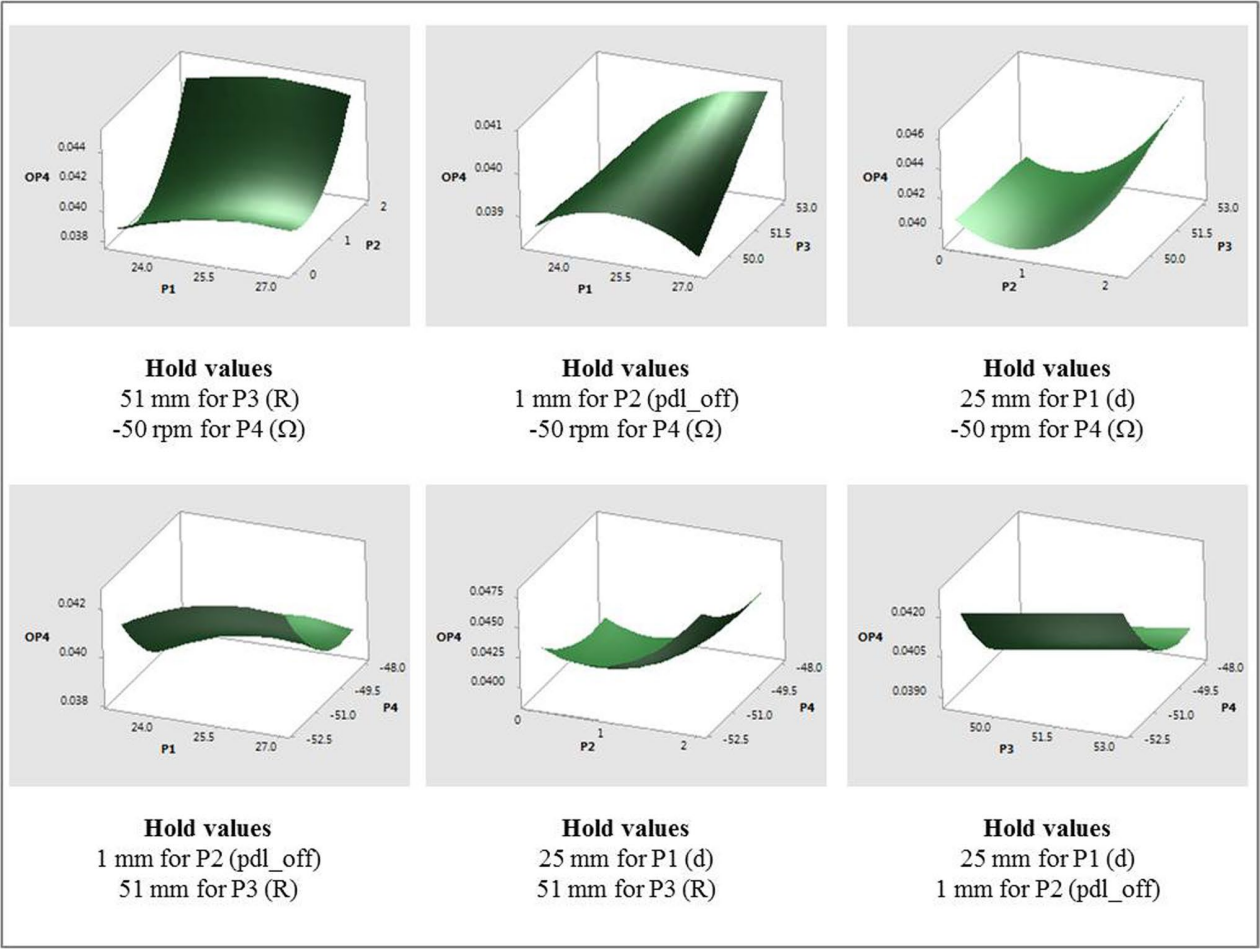
OP4 response surfaces for combinations of various input parameters.

Response surface plots that show the relationship between OP4 and various combinations of all input parameters are shown in Fig. 14. The individual effects of input parameters on the fitted means of OP4, with all other input parameters constant, are as follows:

- The mean values of OP4 were found to change quadratically with P2, but differently than what were observed with OP1 through OP3. OP4 values decreased for approximately 0 ≤ P2 ≤ 0.75 mm and then increased to P2 = 2 mm. One of the interaction effects was between P2 and P3. As with other interaction effects with OP1 through OP3, the mean values of OP4 followed the main effect of P2, with higher values of P3 resulting in higher values of OP4.
- The mean values of OP4 increased and then decreased with increasing P1, increased linearly with P3, and decreased and then increased with increasing values of P4 (magnitude). Another interaction effect was between P1 and P3. While the mean values of OP4 generally increased with increasing values of P1, for the higher radii considered (i.e., P3 = 51 mm and 53 mm), values of OP4 decreased slightly with increasing P1 for P3 = 49 mm.

### CFD Analysis

The individual and combined effects of the input parameters on the overall hydrodynamics in App 2 were analyzed by comparing velocity magnitude vector plots for various parameter combinations. Since the DOE analysis identified the impeller offset to be the most influential parameter, results across cases with different offsets (with all other input parameters constant) are discussed first. Subsequently, results from cases with fixed impeller offset values with other parameters changing are presented.

#### Effect of Impeller Offset

**Fig. 15:**
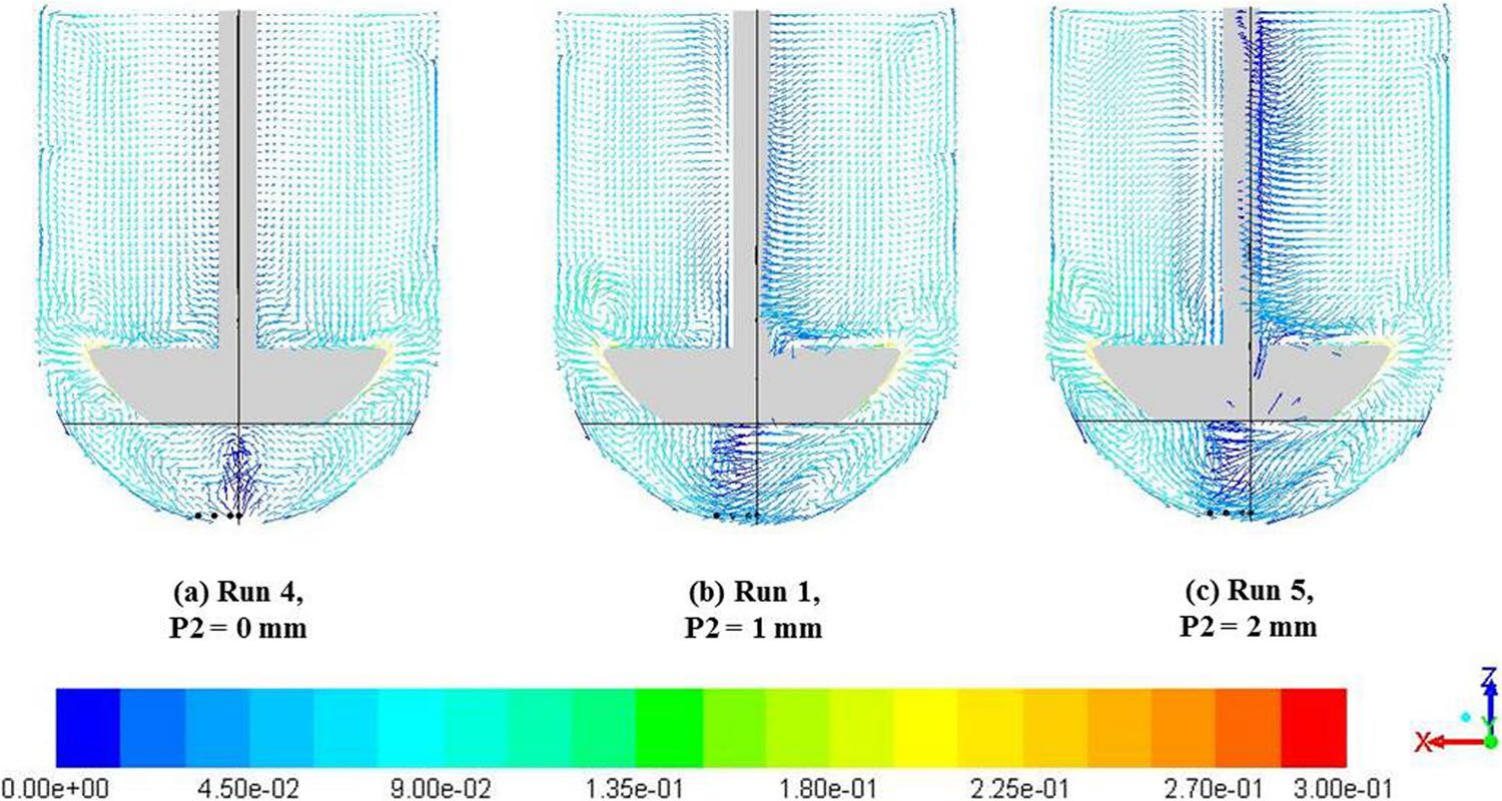
Velocity magnitude (m/s) vectors with varying P2 (pdl_off); P1 (d) = 25 mm, P3 (R) = 51 mm and P4 (Ω) = -50 rpm for all cases.

To understand the effects of impeller offset (P2) as an individual parameter, three cases were identified in which the other input parameters were kept constant. The comparison of velocity magnitude vectors on central vertical planes is shown in Fig. 15. The vertical and horizontal lines in each image represent, respectively, the vessel axis and an axial location that corresponds to P1 (d) = 25 mm. The locations corresponding to each of the output parameters are also shown. The velocity fields for the case with no impeller offset (Fig. 15 (a)) were similar to those seen with the baseline case (Fig. 6).

The immediate effect of the impeller offset was to disturb the symmetry of the velocity fields along the chosen central vertical plane (Fig. 15 (b), (c)). In these cases, the flow at the paddle tips was distributed differently on either side of the paddle. In the region with longer paddle-vessel wall distance the flow was redistributed to form recirculation loops similar to the baseline case. However, in case of shorter paddle-vessel wall distance there is an additional recirculation of flow close to the paddle tip wherein flow was re-directed onto the impeller top surface. Moreover, the expanses of the recirculation areas, on either side of the impeller, were found to adjust to the impeller offset and velocities in the immediate vicinity of the shaft, in the direction opposite to the offset, were found to decrease with increasing impeller offset. More importantly, the low velocity zone is significantly affected by increasing impeller offset values. Specifically, the lowest region of the dead zone was found to be completely disturbed by any impeller offset and was found to have velocity magnitudes greater than zero.

#### Effect of Distance between Vessel and Impeller Bottom Surfaces, Vessel Radius and Impeller Rotational Speed

**Fig. 16:**
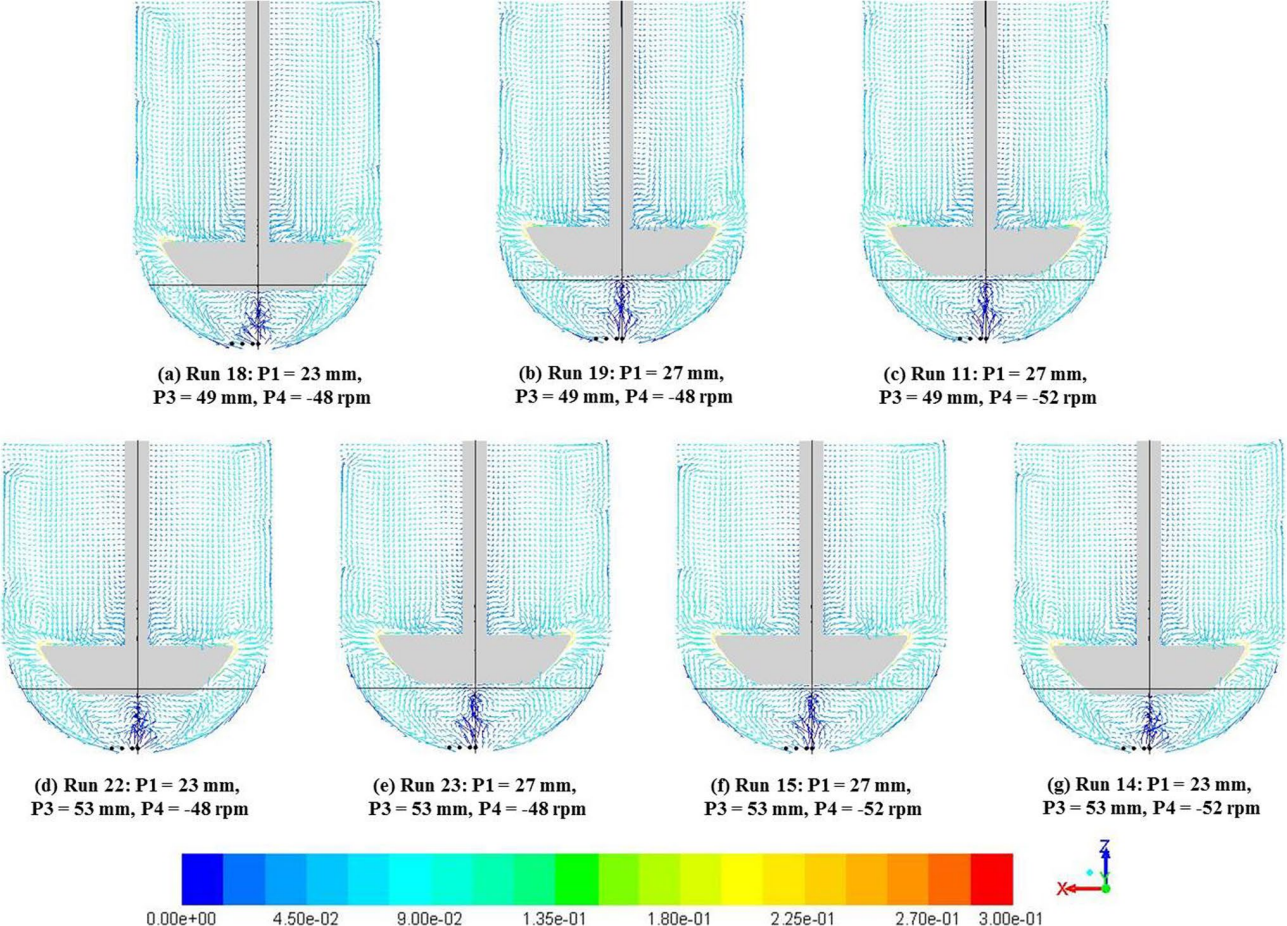
Velocity magnitude (m/s) vectors with varying P1 (d), P3 (R) and P4 (Ω) with P2 (pdl_off) = 0 mm for all cases.

A comparison of velocity magnitude vectors, on central vertical planes, for the seven cases^4^ with no impeller offset is shown in Fig. 16. With symmetry along the plane retained across all these cases, the overall flow characteristics were largely unaffected by changes to the remaining three input parameters being discussed. As with the baseline case, the flow in these cases was characterized by recirculation loops and a low velocity zone below the impeller. It was observed that the dead zone height adjusted to the changes in the distance between vessel and impeller bottom surfaces.

**Fig. 17:**
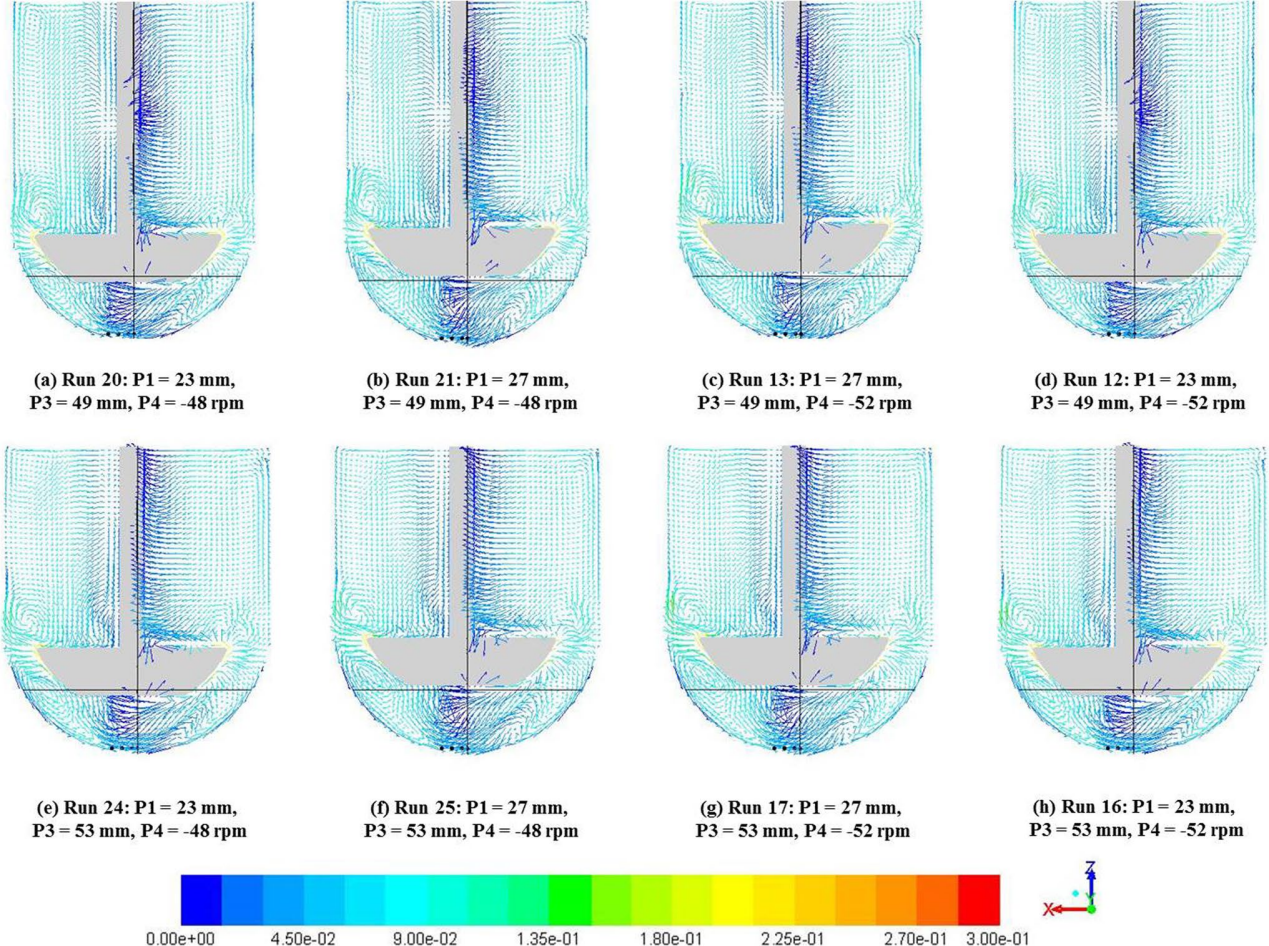
Velocity magnitude (m/s) vectors with varying P1 (d), P3 (R) and P4 (Ω) with P2 (pdl_off) = 2 mm for all cases.

On the other hand, analysis of cases on the other extreme with P2 (pdl_off) = 2 mm confirms the strong effect that impeller offset has on the hydrodynamics in App 2. Vectors for the eight cases with P2 = 2 mm are shown in Fig. 17. The analysis across results of these cases revealed no discernible dominant effects of any other input parameter investigated excepting in expanses of regions with specific flow patterns as arising from changes to geometry. Analysis of Fig. 16 and 17 together indicate that while any changes to the impeller offset lead to significant changes in the hydrodynamics of App 2, the other input parameters investigated do not have the same magnitude of influence on the observed changes compared to the baseline case.

## DISCUSSION

In an ideal setup, where the distance between the vessel and impeller bottom surfaces and the vessel radius are within specifications defined in USP General Chapter < 711 > *Dissolution* (1), and where there is no impeller offset, there are two flow recirculation loops on both sides (one above and another below) of the impeller along a vertical central plane. Additionally, a low velocity magnitude area develops underneath the impeller directly along the impeller axis of rotation. These results are in agreement with those previously reported in literature (7–9).

Several cases with changes to geometric and operational parameters were run as part of the DOE analysis. Of all the parameters considered, the impeller offset which disturbs the geometric symmetry (along the central vertical plane about the impeller axis) was found to have the most dominant effect. Both the symmetric recirculation patterns (above and below the impeller) and the dead zone were disturbed by impeller offset. Even though the variations in input parameters, were combinational in nature, the offset parameter stood out as the parameter of influence. The influence of other parameters, which do not alter the geometric symmetry, on hydrodynamics was limited. These findings are comparable to those of Bai *et al*. (11) who presented the results of CFD studies on the influence of the impeller offset, and the distance between vessel and impeller bottom surfaces as individual parameters.

The influence of the impeller offset was specifically pronounced at locations close to the vessel axis. While the impeller offset was still prominent, contributions of other parameters were found to increase for locations that are farther from the vessel axis and lie outside the bounding edge of the shaft for the baseline case. For these locations the impeller rotation speed was also a significant contributor.

The levels of variation chosen for the input parameters play an important role in the extent of their influence on App 2 hydrodynamics. Even though the span of variation in levels considered for the geometric input parameters were within the compendial tolerances, small changes in impeller offset resulted in bigger influences on all output parameters than for other parameters. Variations considered for the other geometric parameters contributed as influences only for output parameters evaluated at locations that are farther from the vessel axis.

Differences in geometry and/or operational conditions of App 2 can lead to differences in hydrodynamics. From the design space investigated herein, it was seen that even small offsets in impeller location can lead to significant changes in hydrodynamics. On the other hand, changes to impeller rotation speed, vessel radius, and distance between the vessel and impeller bottom surfaces to within specifications in (1), do not alter the hydrodynamics significantly, but do influence the velocity magnitudes at locations below the impeller that are away from the vessel axis.

The model presented is specific to cases where the principal axis of the vessel is parallel to that of the impeller. Other parameters such as impeller/vessel tilt, impeller wobble, vessel irregularities etc. will have to be treated differently from a modeling perspective. All of these can be considered for future work and are not addressed here. The current work is presented as a foundational platform for a basic understanding of how a DOE analysis can be combined with CFD simulations to provide insights into individual and combinational effects of operational and/or design parameters.

## CONCLUSIONS

A DOE study that investigated the individual and two-factor interaction effects of changing four input parameters on four selected output parameters was conducted using CFD simulations. Statistical evaluation of the data generated shows that, of all the input parameters considered, changes to impeller offset has the most dominating effect on all the output parameters. The effect was found not only to be linear but also quadratic in nature as evidenced by the square effects on the output parameters. At locations farthest from the vessel axis, the other three input parameters also contributed to changes in the output parameters. The impeller rotation speed stands out as the second most influential parameter at these two locations. The vessel radius and distance between the vessel and impeller bottom surfaces also have lesser, but still important contributions. Further, interaction effects were found to be low at all locations considered except for the location farthest from the vessel axis.

A comparison of velocity vectors along a central vertical plane across all cases also showed that there was a significant qualitative impact of impeller offset on the overall hydrodynamics in App 2. Any impeller offset led to disturbance of the system symmetry along the vessel axis. Consequently, with any offset, changes were observed to the nature of flow re-distribution from the paddle tips and to the expanse and nature of the dead zone below the impeller. These changes were not observed with any changes to the other input parameters investigated. Even within cases with offset at the highest value changing the other three input parameters did not significantly alter the overall nature of velocity fields. Thus, changes to input parameters within specifications in (1) that did not disturb geometric symmetry were found to have relatively lower influence on the qualitative hydrodynamics of the system.

## Footnotes

1 Literature sources use the terms paddle or impeller to describe the shaft-paddle assembly in App 2. All operational and geometric attributes associated with these terms are understood to apply to both the paddle and the shaft.

2 Some solution strategies employed with the baseline solution (and correspondingly partial results) were previously reported by some of the co-authors [12]

3 The cyclic variation can be attributed to the resolution of very fine flow phenomenon (with low velocity magnitudes) at the location of the output points and the inherent unsteadiness associated with these cases.

4 Run 10, as mentioned previously, did not provide a converged solution and thus was excluded from analysis.

## DISCLAIMERS

Certain commercial equipment, instruments, vendors, or materials are identified in this paper to adequately specify the experimental procedure. Such identification does not imply approval, endorsement, or certification by USP or the authors of the particular brand or product, nor does it imply that the equipment, instrument, vendor, or material is necessarily the best available for the purpose or that any other brand or product was judged to be unsatisfactory or inadequate.
